# Supplementary material for: Genome-Informed Real-Time PCR Assay for Detection of ‘Candidatus Phytoplasma Prunorum,’ Which Is Associated with European Stone Fruit Yellows
Source: Microorganisms. 2025 Apr 17;13(4):929. doi: 10.3390/microorganisms13040929 (PMC12029454; doi:10.3390/microorganisms13040929)
Supplement: Supplementary file 1 [file microorganisms-13-00929-s001.zip › microorganisms-3561890-supplementary.pdf]

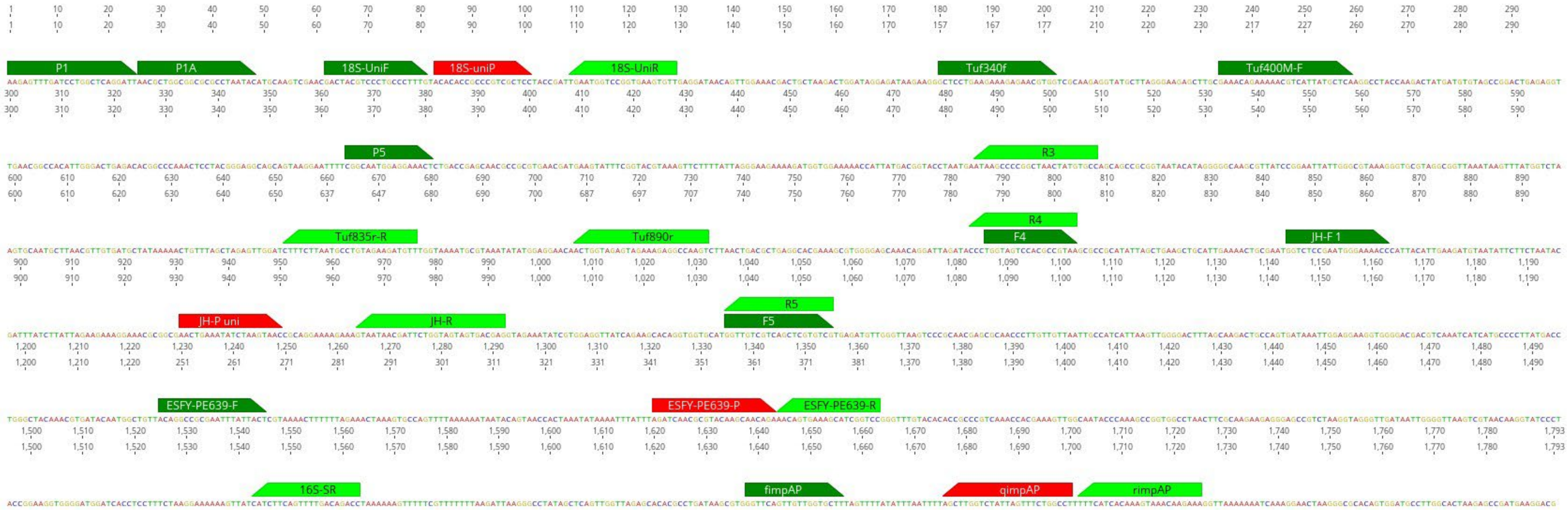

Figure S1. Phytoplasma synthetic gBlocks™ positive control sequence with primer binding site annotations. The gBlocks contains 1,793 bp of the 16S sequence from strawberry lethal yellows phytoplasma (CPA) str. NZSb11 (CP002548). The gBlocks contains all primer binding sites for the 16S semi-nested conventional PCR and associated sequencing primers. The sequence was modified to include the phytoplasma 23S and 18S plant internal control targets for real-time PCR. Additionally, a target within the 16S region was intentionally removed to prevent amplification by Christensen et al. 2004 16S primers. The 23S and 18S markers are located within the 16S sequencing region and provide a unique signature that will allow differentiation from true phytoplasma DNA. Moreover, the gBlocks sequence was modified so that the translated amino acid sequence will read "APHIS SCIENCE" when viewing the first frame; this unique signature is located between the F4 and JHF-1 primers. Due to the extremely low G+C content of the *imp* target for '*Ca.* P. mali,' it was placed in the 3' tail end of the gBlocks outside the sequencing region. The PE639 target for '*Ca.* P. prunorum' also has extremely low G+C content. This target was placed upstream of the 16S-SR primer binding site, within the sequencing region. Primer binding sites for the *tuf* gene were inserted within the 16S sequencing region, after the 18S target and surrounding internal sequencing primer P5 and R3 binding sites.

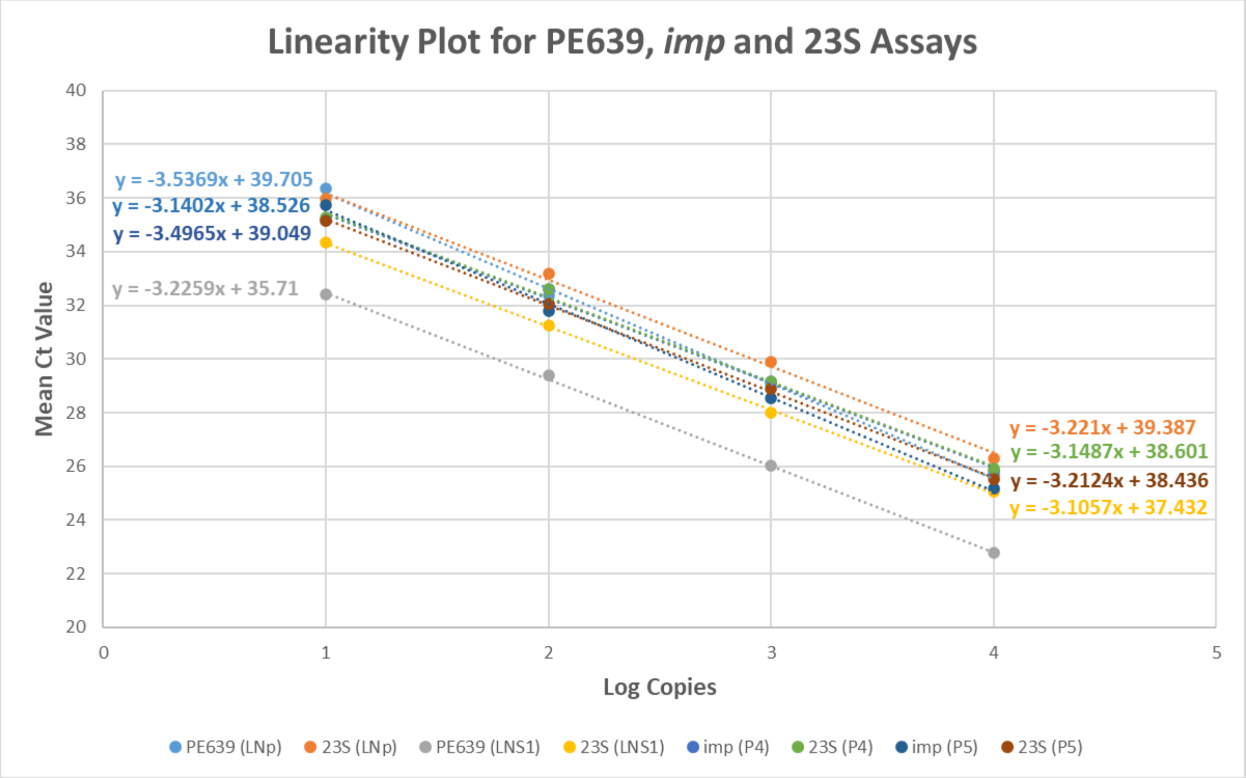

Figure S2. Linear amplification plots for 10-fold serial dilutions of DNA from '*Candidatus* Phytoplasma prunorum' strains LNp and LNS1 and '*Ca.* P. mali' strains P4 and P5. Strains of '*Ca.* P. prunorum' and '*Ca.* P. mali' were each tested with two assays: PE639 and 23S for '*Ca.* P. prunorum', and *imp* and 23S for '*Ca.* P. mali.' Data points represent mean C_t values from three replicates by a single operator on the QuantStudio™ 5 Real-Time PCR System (N=3 per data point). Linear regression equations are displayed.

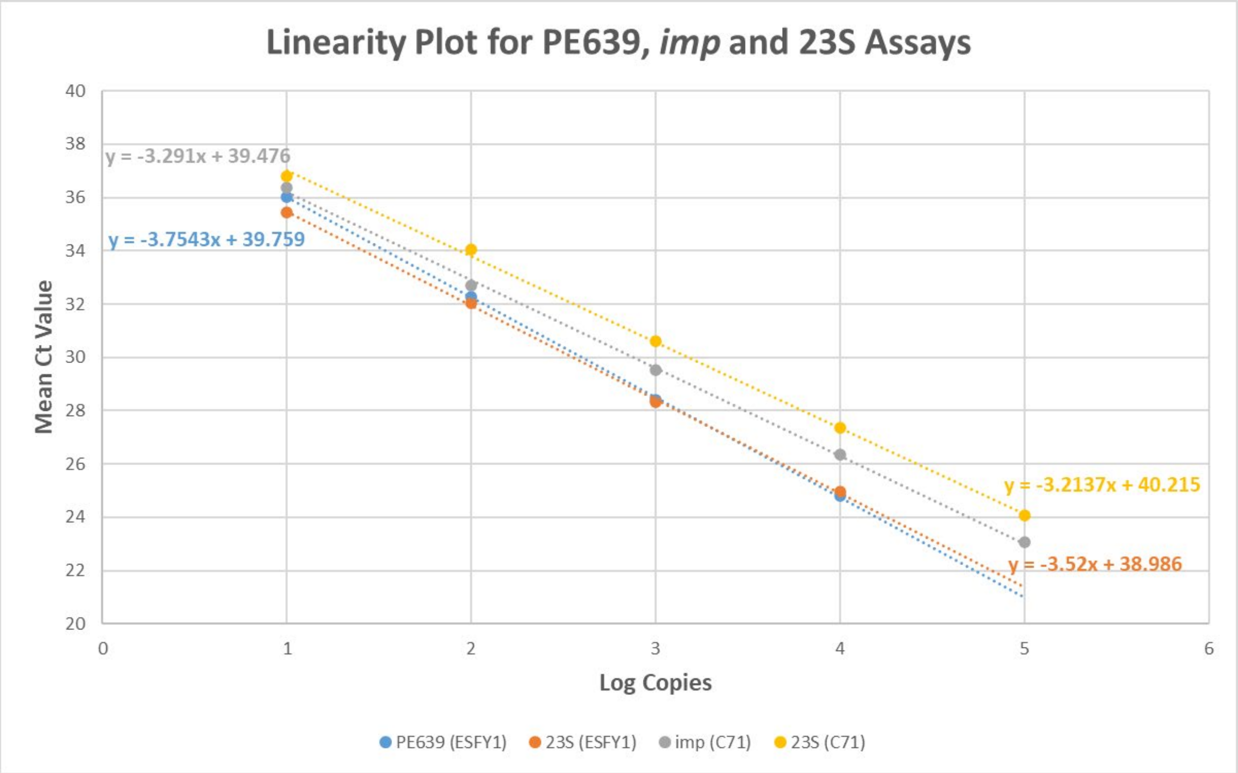

Figure S3. Linear amplification plots for 10-fold serial dilutions from '*Candidatus* Phytoplasma prunorum' strain ESFY1 and '*Ca.* P. mali' strain C71 tested in the CFX OPUS. Strains EFSY1 and C71 were each tested with two assays: PE639 and 23S for ESFY1, and *imp* and 23S for C71. Data points represent mean C_t values from three replicates by a single operator on the CFX96 OPUS Real-Time PCR System (N=3 per data point). Linear regression equations are displayed.

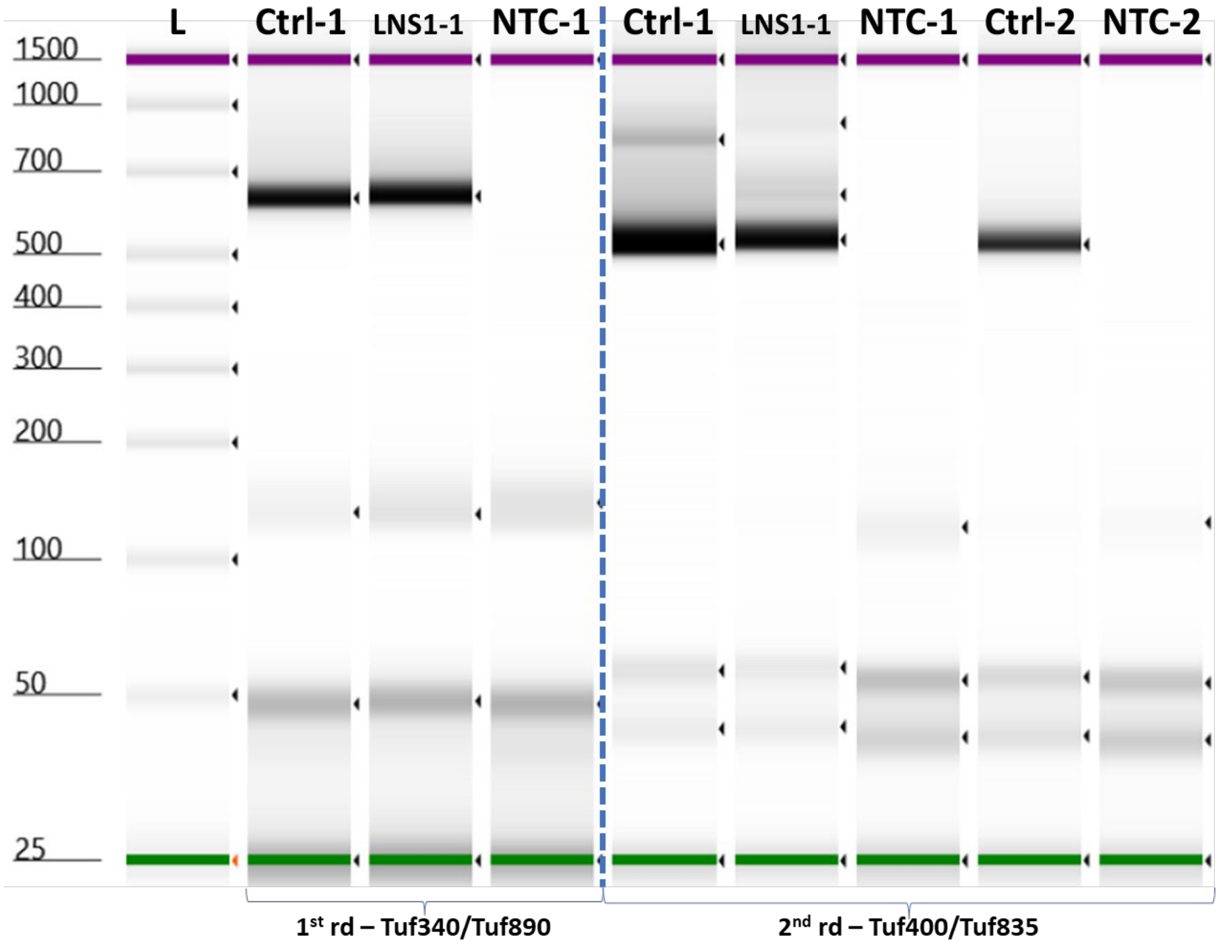

Figure S4. Gel image showing amplicons resulting from nested cPCR targeting the elongation factor Tu (*tuf*) gene of phytoplasmas using *tuf* primer cocktails. The working dilution of the new synthetic gBlocks positive control (**4 fg/µl**) was subjected to nested PCR amplification according to Makarova et al. 2012. The synthetic positive control (**Ctrl**) was tested alongside **LNS1** (‘*Candidatus* Phytoplasma prunorum’) and molecular grade water (**NTC**) as reaction controls. These samples were carried over from 1st round (**-1**) to 2nd round (**-2**). Additionally, the synthetic positive control and an NTC were added into the 2nd round only. Amplicons were analyzed using the 4200 TapeStation System, D1000 ScreenTape, D1000 DNA Ladder (**L**) and D1000 Sample Buffer, according to the manufacturer’s instructions. Data shows that the working dilution (4 fg/µl) produced an amplicon of appropriate size for both rounds of PCR; the second-round product is shorter than the first round. The synthetic positive control worked individually in each round of PCR, as well as across the two rounds of PCR. All reaction controls worked appropriately.

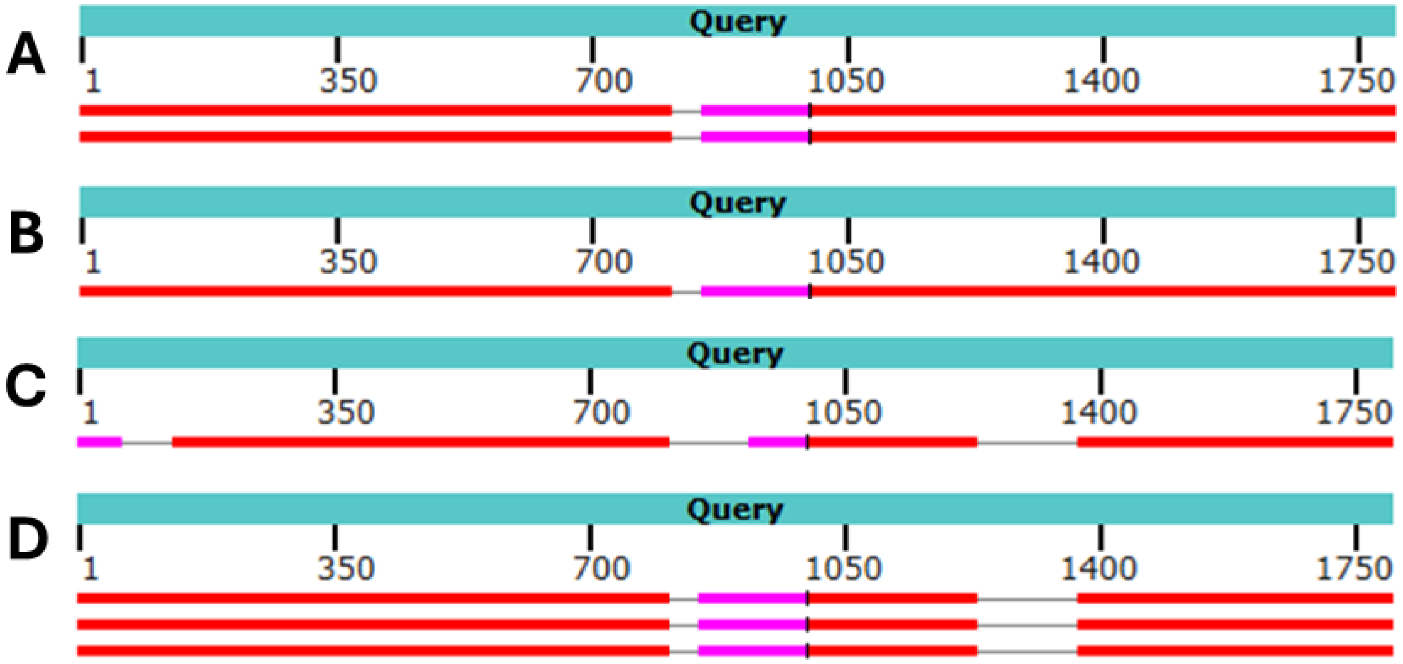

Figure S5. Screenshot showing examples of graphical summaries from NCBI BLAST analysis of the phytoplasma synthetic gBlocks™ positive control. NCBI BLAST analysis revealed highest matches to Strawberry lethal yellows phytoplasma (CPA) str. NZSb11, complete genome (Accession: CP002548) and '*Candidatus* Phytoplasma australiense' complete genome (Accession: AM422018) when using the **A)** core nucleotide (core_nt) database, and highest matches to Strawberry lethal yellows phytoplasma (CPA) str. NZSb11, complete genome (Accession: CP002548) when using the **B)** RefSeq Genome Database (refseq_genomes) and **C)** RefSeq Reference genomes (refseq_reference_genomes). BLAST analysis against **D)** whole-genome shotgun contigs (wgs) database returns highest matches to various phytoplasma. Alignments show gaps where target sequences were inserted and matches to the 23S sequence (usually pink in color), making identifying contamination by the internal control possible by simple BLAST analysis.

Table S1. Elongation factor Tu (*tuf*) DNA barcode primers used in this study.[a]

Primer cocktail	Position in AYWB *tuf* gene	Primer cocktail components	Primer Sequence (5'->3')	Proportion of each primer in the cocktail
Tuf340	157-179	**Tuf340a**	GCTCCTGAAGAAARAGAACGTGG	1:1
		Tuf340b	ACTAAAGAAGAAAAAGAACGTGG	
Tuf400	211-236	**Tuf400aM13F**	GTAAAACGACGGCCAGTGAAACAGAAAAACGTCAYTATGCTCA	1:1:1:1:1
		Tuf400bM13F	GTAAAACGACGGCCAGTGAAACTTCTAAAAGACATTACGCTCA	
		Tuf400cM13F	GTAAAACGACGGCCAGTGAAACATCAAAAAGACAYTATGCTCA	
		Tuf400dM13F	GTAAAACGACGGCCAGTGAAACAGAAAAAAGACAYTATGCTCA	
		Tuf400eM13F	GTAAAACGACGGCCAGTCAAACAGCTAAAAGACATTATYCTCA	
Tuf835	628-654	**Tuf835raT7**	TAATACGACTCACTATAGGGAACATCTTCWACHGGCATTAAGAAAGG	1:1:1
		Tuf835rbT7	TAATACGACTCACTATAGGGAACACCTTCAATAGGCATTAAAAAWGG	
		Tuf835rcT7	TAATACGACTCACTATAGGGAACATCTTCTATAGGTAATAAAAAAGG	
Tuf890	685-710	**Tuf890ra**	ACTTGDCCTCTTTCKACTCTACCAGT	1:1:1
		Tuf890rb	ATTTGTCCTCTTTCWACACGTCCTGT	
		Tuf890rc	ACCATTCCTCTTTCAACACGTCCAGT	

[a]Table recreated from Makarova et al. 2012. Binding sites for primers highlighted in bold letters were added into the synthetic positive control.

Table S2. Nucleotide sequence of the synthetic gBlocks™ positive control for general phytoplasma detection and detection of '*Candidatus* Phytoplasma mali' and '*Ca.* Phytoplasma prunorum.'

PHPL-18Sunictrl-IMP-PE639-tuf_1793bp gBlocks™ positive control
AAGAGTTTGATCCTGGCTCAGGATTAACGCTGGCGGCGCGCCTAATACATGCAAG<br>TCGAACGACTACGTCCCTGCCCTTTGTACACACCGCCCGTCGCTCCTACCGATTGA<br>ATGGTCCGGTGAAGTGTTGAGGATAACAGTTGGAAACGACTGCTAAGACTGGATA<br>GGAGATAAGAAGGGCTCCTGAAGAAAGAGAACGTGGTCGCAAGAGGTATGCTTA<br>GGGAAGAGCTTGCGAAACAGAAAAACGTCATTATGCTCAAGGCCTACCAAGACTA<br>TGATGTGTAGCCGGACTGAGAGGTTGAACGGCCACATTGGGACTGAGACACGGCC<br>CAAACTCCTACGGGAGGCAGCAGTAAGGAATTTTCGGCAATGGAGGAAACTCTGA<br>CCGAGCAACGCCGCGTGAACGATGAAGTATTTCGGTACGTAAAGTTCTTTTATTAG<br>GGAAGAAAAGATGGTGGAAAAACCATTATGACGGTACCTAATGAATAAGCCCCG<br>GCTAACTATGTGCCAGCAGCCGCGGTAATACATAGGGGGCAAGCGTTATCCGGAA<br>TTATTGGGCGTAAAGGGTGCGTAGGCGGTTAAATAAGTTTATGGTCTAAGTGCAA<br>TGCTTAACGTTGTGATGCTATAAAAACTGTTTAGCTAGAGTTGGATCTTTCTTAAT<br>GCCTGTAGAAGATGTTTGGTAAAATGCGTAAATATATGGAGGAACAACTGGTAGA<br>GTAGAAAGAGGCCAAGTCTTAACTGACGCTGAGGCACGAAAGCGTGGGGAGCAA<br>ACAGGATTAGATACCCTGGTAGTCCACGCCGTAAGCGCCGCATATTAGCTGAAGC<br>TGCATTGAAAACTGCGAATGGTCTCCGAATGGGAAAACCCATTACATTGAAGATG<br>TAATATTCTTCTAATACGATTTATCTTATTAGAAGAAAGGAAACGCGGCGAACTG<br>AAATATCTAAGTAACCGCAGGAAAAGAAAGTAATAACGATTCTGGTAGTAGTGAC<br>GAGGTAGAAATATCGTGGAGGTTATCAGAAGCACAGGTGGTGCATGGTTGTCGTC<br>AGCTCGTGTCGTGAGATGTTGGGTTAAGTCCCGCAACGAGCGCAACCCTTGTTGTT<br>AATTGCCATCATTAAGTTGGGGACTTTAGCAAGACTGCCAGTGATAAATTGGAGG<br>AAGGTGGGGACGACGTCAAATCATCATGCCCCTTATGACCTGGGCTACAAACGTG<br>ATACAATGGCTGTTACAGGCCGCGAATTTATTACTCGTAAAACTTTTTTAGAAACT<br>AAAGTGCCAGTTTTAAAAAATAATACAGTAACCACTAAATATAAAATTTATTTAG<br>ATCAACGCGTACAAGCAACAGAAACAGTGAAAGCATCGGTCCGGGTTTGTACACA<br>CCGCCCGTCAAACCACGAAAGTTGGCAATACCCAAAGCCGGTGGCCTAACTTCGC<br>AAGAAGAGGGAGCCGTCTAAGGTAGGGTTGATAATTGGGGTTAAGTCGTAACAA<br>GGTATCCCTACCGGAAGGTGGGGATGGATCACCTCCTTTCTAAGGAAAAAAGTTA<br>TCATCTTCAGTTTTGACAGACCTAAAAAAGTTTTTCGTTTTTTTAAGATTAAGGGC<br>CTATAGCTCAGTTGGTTAGAGCACACGCCTGATAAGCGTGGGTTCAGTTGTTGGTG<br>CTTTAGTTTTATATTTAATTTTAGCTTGGTCTATTAGTTTCTGGCCTTTTTCATCACA<br>AAGTAAACAAGAAAGGTTAAAAAAATCAAAGGAACTAAGGGCGCACAGTGGATG<br>CCTTGGCACTAAGAGCCGATGAAGGACG

Table S3. Limit of detection (LoD) for the PE639 and 23S assays based on testing 10-fold serial dilutions of '*Ca.* P. prunorum' strains ESFY1, LNp and LNS1 in healthy peach (*Prunus persica*) DNA on the QuantStudio™ 5 Real-Time PCR System.

Candidatus **Phytoplasma prunorum' ESFY1**										
	**PE639-FAM**					**23S-FAM**				
**Log Dilution Spike**	**Mean**	**Std Dev**	**CV (%)**	**N**	**% Positive**	**Mean**	**Std Dev**	**CV (%)**	**N**	**% Positive**
0	25.09	0.39	1.55%	9	100.00%	24.87	0.27	1.07%	3	100.00%
1	28.92	0.34	1.17%	9	100.00%	28.76	0.28	0.96%	3	100.00%
2	32.38	0.19	0.60%	9	100.00%	32.28	0.22	0.67%	3	100.00%
3	35.81	0.39	1.09%	9	100.00%	35.55	0.10	0.28%	3	100.00%
4	38.17	0.97	-	9	33.33%	39.75	0.11	0.27%	3	66.67%
5	-	-	-	9	0.00%	-	-	-	3	0.00%
Candidatus **Phytoplasma prunorum' LNP**										
	**PE639-FAM**					**23S-FAM**				
**Log Dilution Spike**	**Mean**	**Std Dev**	**CV (%)**	**N**	**% Positive**	**Mean**	**Std Dev**	**CV (%)**	**N**	**% Positive**
0	25.66	0.02	0.07%	3	100.00%	26.32	0.07	0.25%	3	100.00%
1	29.06	0.05	0.16%	3	100.00%	29.89	0.23	0.76%	3	100.00%
2	32.38	0.16	0.49%	3	100.00%	33.18	0.23	0.70%	3	100.00%
3	36.35	0.55	1.51%	3	100.00%	35.96	0.61	1.70%	3	100.00%
4	37.49	-	-	3	33.33%	38.45	-	-	3	33.33%
5	-	-	-	3	0.00%	-	-	-	3	0.00%
Candidatus **Phytoplasma prunorum' LNS1**										
	**PE639-FAM**					**23S-FAM**				
**Log Dilution Spike**	**Mean**	**Std Dev**	**CV (%)**	**N**	**% Positive**	**Mean**	**Std Dev**	**CV (%)**	**N**	**% Positive**
0	22.76	0.20	0.87%	3	100.00%	25.07	0.21	0.84%	3	100.00%
1	26.03	0.11	0.43%	3	100.00%	28.01	0.12	0.44%	3	100.00%
2	29.40	0.14	0.48%	3	100.00%	31.25	0.15	0.48%	3	100.00%
3	32.39	0.26	0.80%	3	100.00%	34.35	0.23	0.66%	3	100.00%
4	35.98	0.73	2.04%	3	100.00%	37.85	0.27	0.71%	3	66.67%
5	37.63	-	-	3	33.33%	-	-	-	3	0.00%

Mean = average cycle threshold (C_t) value
Std Dev = standard deviation
CV = coefficient of variation (Std Dev / Mean)

Table S4. Limit of detection (LoD) for the PE639 and 23S assays based on testing 10-fold serial dilutions of '*Ca.* P. prunorum' strain ESFY1 in healthy peach (*Prunus persica*) DNA on the CFX96 OPUS Real-Time PCR Detection System.

		PE639-FAM				23S-FAM			
**Log Dilution**	**N**	**Mean**	**Std Dev**	**CV (%)**	**% Positive**	**Mean**	**Std Dev**	**CV (%)**	**% Positive**
0	3	24.79	0.40	1.60%	100.00%	24.95	0.22	0.86%	100.00%
1	3	28.42	0.14	0.49%	100.00%	28.31	0.12	0.42%	100.00%
2	3	32.26	0.06	0.18%	100.00%	32.03	0.02	0.06%	100.00%
3	3	36.02	0.71	1.98%	100.00%	35.45	0.15	0.42%	100.00%
4	3	37.53	-	-	33.33%	37.97	0.34	0.90%	66.67%
5	3	-	-	-	0.00%	-	-	-	0.00%

Mean = Average cycle threshold (C_t) value
Std Dev = standard deviation
CV = coefficient of variation (Std Dev / Mean)

Table S5. Limit of detection (LoD) for the PE639 and 23S assays based on testing 10-fold serial dilutions of '*Ca.* P. prunorum' strains ESFY1, LNp and LNS1 in different host backgrounds on the QuantStudio™ 5 Real-Time PCR System.

			PE639-FAM												23S-FAM			
			ESFY1 (Dilution 3)				LNp (Dilution 3)				LNS1 (Dilution 4)				LNS1 (Dilution 3)			
**Host**	**Host (Common)**	**N**	**Mean**	**Std Dev**	**CV (%)**	**% Positive**	**Mean**	**Std Dev**	**CV (%)**	**% Positive**	**Mean**	**Std Dev**	**CV (%)**	**% Positive**	**Mean**	**Std Dev**	**CV (%)**	**% Positive**
Prunus persica	Peach	3	35.46	0.58	1.65%	100.00%	36.35	0.55	1.51%	100.00%	35.98	0.73	2.04%	100.00%	34.35	0.23	0.66%	100.00%
Prunus armeniaca	Apricot	3	35.31	0.31	0.88%	100.00%	35.96	0.67	1.86%	100.00%	36.60	1.18	3.23%	100.00%	35.69	0.80	2.24%	100.00%
Prunus salicina	Japanese plum	3	35.09	0.69	1.96%	100.00%	36.19	0.29	0.80%	100.00%	35.15	0.40	1.13%	100.00%	34.93	0.40	1.15%	100.00%
Prunus avium	Cherry	3	35.04	0.41	1.18%	100.00%	36.11	1.83	5.07%	100.00%	36.35	0.99	2.72%	100.00%	34.89	0.09	0.25%	100.00%

Mean = Average cycle threshold (C_t) value
Std Dev = standard deviation
CV = coefficient of variation (Std Dev / Mean)

Table S6. Real-time PCR results for an inclusivity/exclusivity screen to determine assay specificity and selectivity (positive/negative).

Organism	Sample ID	DNA Source	Prunorum Assay		WI-B-T-1-116 (mali)		WI-B-T-1-66 (Phpl)	
			PE639	18S	*imp*	18S	23S	18S
Candidatus Phytoplasma'	GY-U	Infected plant material	-	+	-	+	+	+
Candidatus Phytoplasma'	ALY	Infected plant material	-	+	-	+	+	+
Candidatus Phytoplasma'	AY-A	Infected plant material	-	+	-	+	+	+
Candidatus Phytoplasma'	CoPh	Infected plant material	-	+	-	+	+	+
Candidatus Phytoplasma'	BVK	Infected plant material	-	+	-	+	+	+
Candidatus Phytoplasma'	PEY	Infected plant material	-	+	-	+	+	+
Candidatus Phytoplasma asteris'	23092601-01	Infected plant material	-	+	-	+	+	+
Candidatus Phytoplasma asteris'	23092601-02	Infected plant material	-	+	-	+	+	+
Candidatus Phytoplasma asteris'	23092601-03	Infected plant material	-	+	-	+	+	+
Candidatus Phytoplasma aurantifolia'	WBDL	Infected plant material	-	+	-	+	+	+
Candidatus Phytoplasma brasilliense'	SuV	Infected plant material	-	+	-	+	+	+
Candidatus Phytoplasma mali'	P4	Infected plant material	-	+	+	+	+	+
Candidatus Phytoplasma mali'	P6	Infected plant material	-	+	+	+	+	+
Candidatus Phytoplasma mali'	AP-15	Infected plant material	+[a]	+	+	+	+	+
Candidatus Phytoplasma mali'	APxN	Infected plant material	+[a]	+	+	+	+	+
Candidatus Phytoplasma mali'	AP-1	Infected plant material	-	+	+	+	+	+
Candidatus Phytoplasma mali'	AP-2	Infected plant material	-	+	+	+	+	+
Candidatus Phytoplasma mali'	AP-3	Infected plant material	-	+	+	+	+	+
Candidatu s Phytoplasma pini'-related	MDPP	Infected plant material	-	+	-	+	+	+
Candidatus Phytoplasma prunorum'	ESFY1	Infected plant material	+	+	-	+	+	+
Candidatus Phytoplasma prunorum'	P1	Infected plant material	+	+	-	+	+	+
Candidatus Phytoplasma prunorum'	LNp	Infected plant material	+	+	-	+	+	+
Candidatus Phytoplasma prunorum'	LNS1	Infected plant material	+	+	-	+	+	+
Candidatus Phytoplasma prunorum'	LNS2	Infected plant material	+	+	-	+	+	+
Candidatus Phytoplasma prunorum'	ESFY-1A	Infected plant material	+	+	-	+	+	+
Candidatus Phytoplasma prunorum'	ESFY-2A	Infected plant material	+	+	-	+	+	+
Candidatus Phytoplasma prunorum'	ESFY-1P	Infected plant material	+	+	-	+	+	+
Candidatus Phytoplasma prunorum'	ESFY-2P	Infected plant material	+	+	-	+	+	+
Candidatus Phytoplasma prunorum'	ESFY-1PE	Infected plant material	+	+	-	+	+	+
Candidatus Phytoplasma prunorum'	ESFY-2PE	Infected plant material	+	+	-	+	+	+
Candidatus Phytoplasma palmae'	19101702-01	Infected plant material	-	+	-	+	+	+
Candidatus Phytoplasma palmae'	19110501-02	Infected plant material	-	+	-	+	+	+
Candidatus Phytoplasma pruni' rrnA	19122001-22	Infected plant material	-	+	-	+	+	+
Candidatus Phytoplasma pruni' rrnA	19122001-41	Infected plant material	-	+	-	+	+	+
Candidatus Phytoplasma pruni' rrnA	20012301-04	Infected plant material	-	+	-	+	+	+
Candidatus Phytoplasma pruni' rrnA	20012301-05	Infected plant material	-	+	-	+	+	+
Candidatus Phytoplasma pruni' rrnA	20012301-06	Infected plant material	-	+	-	+	+	+
Candidatus Phytoplasma pruni' rrnA	20012301-07	Infected plant material	-	+	-	+	+	+
Candidatus Phytoplasma pruni' rrnA	20012301-08	Infected plant material	-	+	-	+	+	+
Candidatus Phytoplasma pruni' rrnA	20012301-09	Infected plant material	-	+	-	+	+	+
Candidatus Phytoplasma pruni' rrnA	20012301-10	Infected plant material	-	+	-	+	+	+
Candidatus Phytoplasma pruni' rrnA	20012301-11	Infected plant material	-	+	-	+	+	+
Candidatus Phytoplasma pruni' rrnA	21021201-02	Infected plant material	-	+	-	+	+	+
Candidatus Phytoplasma pruni' rrnA	21021201-03	Infected plant material	-	+	-	+	+	+
Candidatus Phytoplasma pyri'	20012301-03	Infected plant material	-	+	-	+	+	+
Candidatus Phytoplasma pyri'	21021201-01	Infected plant material	-	+	-	+	+	+
Candidatus Phytoplasma pyri'	23111702-01	Infected plant material	-	+	-	+	+	+
Candidatus Phytoplasma pyri'	P3	Infected plant material	-	+	-	+	+	+
Candidatus Phytoplasma pyri'	PD	Infected plant material	-	+	-	+	+	+
Candidatus Phytoplasma rubi'	RuS	Infected plant material	-	+	-	+	+	+
Candidatus Phytoplasma solani'	CH-1	Infected plant material	-	+	-	+	+	+
Candidatus Phytoplasma solani'	LNIV	Infected plant material	-	+	-	+	+	+
Candidatus Phytoplasma vitis'-related	19041105-01	Infected plant material	-	+	-	+	+	+
Candidatus Phytoplasma vitis'-related	19122001-01	Infected plant material	-	+	-	+	+	+
Candidatus Phytoplasma vitis'-related	19122001-26	Infected plant material	-	+	-	+	+	+
Candidatus Phytoplasma vitis'-related	19122001-32	Infected plant material	-	+	-	+	+	+
Candidatus Phytoplasma vitis'-related	19122001-40	Infected plant material	-	+	-	+	+	+
Candidatus Phytoplasma vitis'-related	19122001-42	Infected plant material	-	+	-	+	+	+
Candidatus Phytoplasma vitis'-related	21031201-01	Infected plant material	-	+	-	+	+	+
Candidatus Phytoplasma vitis'-related	21031201-02	Infected plant material	-	+	-	+	+	+
Candidatus Phytoplasma vitis'-related	21042602-01	Infected plant material	-	+	-	+	+	+
Candidatus Phytoplasma vitis'-related	21042602-02	Infected plant material	-	+	-	+	+	+
Candidatus Phytoplasma vitis'- related	21012601-01	Infected plant material	-	+	-	+	+	+
Candidatus Phytoplasma vitis'- related	21012601-02	Infected plant material	-	+	-	+	+	+
Candidatus Phytoplasma vitis'- related	19081401-01	Infected plant material	-	+	-	+	+	+
Fragaria × ananassa	Strawberry	Healthy plant material	-	+	-	+	-	+
Fragaria × ananassa	Strawberry (Fruit)	Healthy plant material	-	+	-	+	-	+
Malus domestica	Apple	Healthy plant material	-	+	-	+	-	+
Prunus armeniaca	Apricot	Healthy plant material	-	+	-	+	-	+
Prunus avium	Cherry	Healthy plant material	-	+	-	+	-	+
Prunus persica	Peach	Healthy plant material	-	+	-	+	-	+
Prunus persica	Peach	Healthy plant material	-	+	-	+	-	+
Prunus salicina	Japanese plum	Healthy plant material	-	+	-	+	-	+
Prunus sp.	Plum	Healthy plant material	-	+	-	+	-	+
Pyrus communis	Pear	Healthy plant material	-	+	-	+	-	+
Pyrus pyrifolia var. Kosui	Asian pear	Healthy plant material	-	+	-	+	-	+
Pyrus pyrifolia var. Kumoi	PI 228015	Healthy plant material	-	+	-	+	-	+

Organism	Sample ID	DNA Source	Prunorum Assay		WI-B-T-1-116 (mali)		WI-B-T-1-66 (Phpl)	
			PE639	18S	*imp*	18S	23S	18S
Pyrus pyrifolia var. Hayatama	Asian pear	Healthy plant material	-	+	-	+	-	+
Pyrus pyrifolia var. Tama	Asian pear	Healthy plant material	-	+	-	+	-	+
Pyrus ussuriensis var. China	PI 542009	Healthy plant material	-	+	-	+	-	+
Pyrus ussuriensis var. Mien Suan Li	PI 542006	Healthy plant material	-	+	-	+	-	+
Aureobasidium pullulans	DSM 14940	Culture	-	-	-	-	-	-
Aureobasidium pullulans	DSM 14941	Culture	-	-	-	-	-	-
Erwinia billingae	Eh24	Culture	-	+	-	+	-	+
Pantoea agglomerans	E325	Culture	-	-	-	-	-	-
Pantoea vagans	C9-1	Culture	-	-	-	-	-	-
Pseudomonas fluorescens	A506	Culture	-	-	-	+	-	-
Alternaria alternata	22033001-01 (B)	Culture	-	+	-	+	-	+
Colletotrichum gloeospiriodes	22033001-01 (C)	Culture	-	-	-	+	-	-
Colletotrichum gloeospiriodes	22033001-01 (D1)	Culture	-	-	-	+	-	-
Colletotrichum queenslandicum	17030501 (C-5)	Culture	-	+	-	+	-	+
Colletotrichum queenslandicum	17030501 (C-11)	Culture	-	+	-	+	-	+
Erwinia amylovora	Fire blight	Culture	-	-	-	-	-	-
Erwinia amylovora	Fire blight	Infected plant material	-	+	-	+	-	+
Erwinia amylovora	Ea110	Culture	-	-	-	-	-	-
Erwinia amylovora	Ea153	Culture	-	-	-	-	-	-
Erwinia amylovora	Parkdale	Culture	-	-	-	-	-	-
Erwinia aphidicola	17110702-04	Culture	-	-	-	-	-	-
Erwinia billingae	NCPPB 661[T]	Culture	-	-	-	-	-	-
Erwinia persicina	LA611	Culture	-	+	-	+	-	+
Erwinia persicina	LA659	Culture	-	-	-	-	-	-
Erwinia persicina	19122706-01	Culture	-	-	-	-	-	-
Erwinia piriflorinigrans	CFBP 5888[T]	Culture	-	-	-	-	-	-
Erwinia pyrifoliae	Ep 28/96	Culture	-	-	-	-	-	-
Erwinia pyrifoliae	Ep 4/97	Culture	-	-	-	-	-	-
Erwinia pyrifoliae	Ejp 556	Culture	-	+	-	-	-	+
Erwinia pyrifoliae	Ejp 617	Culture	-	+	-	-	-	+
Erwinia pyrifoliae	23-02275	Culture	-	+	-	+	-	+
Erwinia rhapontici	19122702-01	Culture	-	-	-	-	-	-
Erwinia tasmaniensis	Et1/99[T]	Culture	-	-	-	-	-	-
Erwinia uzenensis	NCPPB 4475[T]	Culture	-	-	-	-	-	-
Monilinia fructicola	19090401-01A	Infected plant material	-	+	-	+	-	+
Monilinia fructicola	19090401-02A	Infected plant material	-	+	-	+	-	+
Monilinia fructicola	Mf35	Culture	-	+	-	+	-	+
Monilinia fructigena	Mfg2-GE-A E	Culture	-	+	-	-	-	+
Monilinia laxa	PSG1	Culture	-	-	-	-	-	-
Monilinia polystroma	SP61	Culture	-	-	-	-	-	-
Neofabraea alba	PD-1696	Infected plant material	-	+	-	+	-	+
Neofabraea sp.	PD-1597	Infected plant material	-	+	-	+	-	+
Neofabraea sp.	PD-1617	Infected plant material	-	+	-	+	-	+
Neofabraea sp.	PD-1655B	Infected plant material	-	+	-	+	-	+
Pantoea agglomerans	23091801-01	Culture	-	-	-	-	-	-
Pantoea agglomerans	23091801-03	Culture	-	-	-	-	-	-
Pantoea agglomerans	23091801-04	Culture	-	-	-	-	-	-
Pantoea allii	23091801-02	Culture	-	-	-	-	-	-
Pantoea ananatis	E	Culture	-	+	-	+	-	+
Pantoea ananatis	CES-5 (JM-55)	Culture	-	+	-	+	-	+
Pantoea ananatis	CES-14 (SM-272)	Culture	-	+	-	+	-	+
Pantoea stewartii	DCop3-07	Culture	-	+	-	+	-	+
Pantoea stewartii	PP685	Culture	-	+	-	+	-	+
Phacidiopycnis wasingtonensis	PD-1597	Infected plant material	-	+	-	+	-	+
Phacidiopycnis wasingtonensis	PD-1655B	Infected plant material	-	+	-	+	-	+
Pseudomonas syringae	JL2583	Culture	-	-	-	-	-	-
Sphaeropsis pyriputrescens	PD-1655B	Infected plant material	-	+	-	+	-	+
Venturia inaequalis	Apple Scab	Infected plant material	-	+	-	+	-	+
Xanthomonas arboricola pv. *corylina*	JL2611	Culture	-	-	-	-	-	-
Xyllela fastidiosa	XFS 253	Infected plant material	-	+	-	+	-	+
Xyllela fastidiosa	XFS 254	Infected plant material	-	+	-	+	-	+
Xyllela fastidiosa	XFS 946	Infected plant material	-	+	-	+	-	+
Xyllela fastidiosa subsp. *multiplex*	Peach Texas A&M (1)	Infected plant material	-	+	-	+	-	+
Xyllela fastidiosa subsp. *multiplex*	Peach Leaves Georgia	Infected plant material	-	+	-	+	-	+

[a]Late positive amplification with PE639 was observed with two strains of '*Candidatus* Phytoplasma mali' (AP-15 and APxN). Amplicon sequencing was used to determine the false results were from contamination. 16S amplicons were sequenced using MinION (Oxford Nanopore Technologies, Oxford, UK). Reads were imported into Geneious Prime and used to make a custom BLAST database. 16S sequence from the '*Ca*. P. prunorum' reference (AJ542544) was used as the query to identify reads from '*Ca*. P. prunorum.' Analyses revealed the presence of '*Ca*. P. prunorum' within these samples, suggesting contamination or coinfection. Approximately three and fifteen reads were found in the AP-15 and APxN extracts, respectively. While no direct conversion between read number and C_t value can be made, data demonstrated the appropriate correlation between read number and relative C_t value; APxN extract contained more reads and produced a lower relative C_t value that AP-15, corresponding to increased contamination.

Table S7. Real-time PCR results for an inclusivity/exclusivity screen to determine assay specificity and selectivity (C_t value).

Organism	Sample ID	DNA Source	Prunorum Assay		WI-B-T-1-116 (mali)		WI-B-T-1-66 (Phpl)	
			PE639	18S	*imp*	18S	23S	18S
Candidatus Phytoplasma'	GY-U	Infected plant material	-	16.20	-	16.11	20.59	16.15
Candidatus Phytoplasma'	ALY	Infected plant material	-	18.64	-	18.46	21.67	18.68
Candidatus Phytoplasma'	AY-A	Infected plant material	-	16.21	-	15.84	18.58	16.45
Candidatus Phytoplasma'	CoPh	Infected plant material	-	14.65	-	14.43	18.04	14.88
Candidatus Phytoplasma'	BVK	Infected plant material	-	22.49	-	22.61	25.59	21.96
Candidatus Phytoplasma'	PEY	Infected plant material	-	15.86	-	15.19	22.15	15.51
Candidatus Phytoplasma asteris'	23092601-01	Infected plant material	-	18.53	-	18.87	34.41	20.78
Candidatus Phytoplasma asteris'	23092601-02	Infected plant material	-	31.56	-	31.56	35.83	35.11
Candidatus Phytoplasma asteris'	23092601-03	Infected plant material	-	18.64	-	18.34	33.83	18.86
Candidatus Phytoplasma aurantifolia'	WBDL	Infected plant material	-	14.55	-	14.11	17.04	14.08
Candidatus Phytoplasma brasilliense'	SuV	Infected plant material	-	13.96	-	13.76	18.43	14.10
Candidatus Phytoplasma mali'	P4	Infected plant material	-	19.34	25.42	19.10	25.55	20.12
Candidatus Phytoplasma mali'	P6	Infected plant material	-	18.72	25.05	18.66	25.33	19.39
Candidatus Phytoplasma mali'	AP-15	Infected plant material	**36.00**[a]	14.21	20.08	13.88	20.98	13.80
Candidatus Phytoplasma mali'	APxN	Infected plant material	**33.16**[a]	14.44	21.26	13.82	22.01	13.89
Candidatus Phytoplasma mali'	AP-1	Infected plant material	-	16.34	26.63	15.18	21.92	15.63
Candidatus Phytoplasma mali'	AP-2	Infected plant material	-	16.09	23.15	15.02	18.90	15.80
Candidatus Phytoplasma mali'	AP-3	Infected plant material	-	18.91	27.39	18.19	21.98	18.98
Candidatu s Phytoplasma pini'-related	MDPP	Infected plant material	-	12.50	-	12.47	24.49	12.45
Candidatus Phytoplasma prunorum'	ESFY1	Infected plant material	27.52	20.76	-	20.49	27.46	20.96
Candidatus Phytoplasma prunorum'	P1	Infected plant material	25.28	18.22	-	18.27	26.57	19.98
Candidatus Phytoplasma prunorum'	LNp	Infected plant material	21.57	12.34	-	11.94	21.55	12.45
Candidatus Phytoplasma prunorum'	LNS1	Infected plant material	19.01	15.22	-	14.25	19.98	14.19
Candidatus Phytoplasma prunorum'	LNS2	Infected plant material	18.35	14.70	-	15.22	20.67	14.98
Candidatus Phytoplasma prunorum'	ESFY-1A	Infected plant material	17.83	13.87	-	13.81	20.55	14.01
Candidatus Phytoplasma prunorum'	ESFY-2A	Infected plant material	15.83	12.45	-	12.47	19.05	12.49
Candidatus Phytoplasma prunorum'	ESFY-1P	Infected plant material	20.77	15.70	-	15.62	23.20	15.73
Candidatus Phytoplasma prunorum'	ESFY-2P	Infected plant material	21.32	16.93	-	16.71	23.35	16.97
Candidatus Phytoplasma prunorum'	ESFY-1PE	Infected plant material	23.38	18.74	-	18.59	25.13	18.68
Candidatus Phytoplasma prunorum'	ESFY-2PE	Infected plant material	22.90	19.08	-	18.78	24.12	18.94
Candidatus Phytoplasma palmae'	19101702-01	Infected plant material	-	17.07	-	16.40	23.38	15.15
Candidatus Phytoplasma palmae'	19110501-02	Infected plant material	-	14.49	-	15.25	25.30	13.57
Candidatus Phytoplasma pruni' rrnA	19122001-22	Infected plant material	-	14.99	-	15.51	23.98	13.62
Candidatus Phytoplasma pruni' rrnA	19122001-41	Infected plant material	-	17.56	-	17.85	25.72	15.95
Candidatus Phytoplasma pruni' rrnA	20012301-04	Infected plant material	-	16.90	-	17.09	28.53	16.72
Candidatus Phytoplasma pruni' rrnA	20012301-05	Infected plant material	-	18.63	-	19.07	27.65	18.89
Candidatus Phytoplasma pruni' rrnA	20012301-06	Infected plant material	-	16.40	-	16.83	27.89	16.96
Candidatus Phytoplasma pruni' rrnA	20012301-07	Infected plant material	-	17.75	-	18.39	28.98	17.79
Candidatus Phytoplasma pruni' rrnA	20012301-08	Infected plant material	-	16.14	-	16.55	25.38	16.15
Candidatus Phytoplasma pruni' rrnA	20012301-09	Infected plant material	-	17.62	-	17.86	25.39	17.89
Candidatus Phytoplasma pruni' rrnA	20012301-10	Infected plant material	-	16.94	-	17.32	23.58	16.90
Candidatus Phytoplasma pruni' rrnA	20012301-11	Infected plant material	-	16.91	-	17.07	22.49	16.86
Candidatus Phytoplasma pruni' rrnA	21021201-02	Infected plant material	-	13.19	-	13.51	23.99	15.93
Candidatus Phytoplasma pruni' rrnA	21021201-03	Infected plant material	-	13.45	-	13.37	24.12	15.93
Candidatus Phytoplasma pyri'	P3	Infected plant material	-	21.56	-	20.66	31.38	22.81
Candidatus Phytoplasma pyri'	PD	Infected plant material	-	20.68	-	20.35	25.26	19.91
Candidatus Phytoplasma pyri'	20012301-03	Infected plant material	-	15.40	-	15.49	23.18	15.31
Candidatus Phytoplasma pyri'	21021201-01	Infected plant material	-	15.64	-	15.98	28.99	20.07
Candidatus Phytoplasma pyri'	23111702-01	Infected plant material	-	14.45	-	14.34	27.91	13.94
Candidatus Phytoplasma rubi'	RuS	Infected plant material	-	14.03	-	13.84	19.02	13.39
Candidatus Phytoplasma solani'	CH-1	Infected plant material	-	16.53	-	16.62	20.25	17.12
Candidatus Phytoplasma solani'	LNIV	Infected plant material	-	15.29	-	14.76	20.28	14.84
Candidatus Phytoplasma vitis'-related	19041105-01	Infected plant material	-	21.32	-	21.94	23.27	22.34
Candidatus Phytoplasma vitis'-related	19122001-01	Infected plant material	-	13.28	-	13.90	21.49	12.45
Candidatus Phytoplasma vitis'-related	19122001-26	Infected plant material	-	14.72	-	14.99	36.91	13.50
Candidatus Phytoplasma vitis'-related	19122001-32	Infected plant material	-	12.22	-	12.40	35.94	11.07
Candidatus Phytoplasma vitis'-related	19122001-40	Infected plant material	-	18.79	-	18.97	23.58	17.07
Candidatus Phytoplasma vitis'-related	19122001-42	Infected plant material	-	13.81	-	13.99	18.11	12.85
Candidatus Phytoplasma vitis'-related	21031201-01	Infected plant material	-	10.11	-	15.05	38.08	18.77
Candidatus Phytoplasma vitis'-related	21031201-02	Infected plant material	-	10.25	-	15.05	36.32	16.93
Candidatus Phytoplasma vitis'-related	21042602-01	Infected plant material	-	13.71	-	13.96	?	19.40
Candidatus Phytoplasma vitis'-related	21042602-02	Infected plant material	-	13.81	-	14.45	?	18.96
Candidatus Phytoplasma vitis'- related	21012601-01	Infected plant material	-	13.32	-	13.55	19.37	13.78
Candidatus Phytoplasma vitis'- related	21012601-02	Infected plant material	-	16.58	-	17.07	21.98	15.86
Candidatus Phytoplasma vitis'- related	19081401-01	Infected plant material	-	12.08	-	12.39	19.54	10.37
Fragaria × ananassa	Strawberry	Healthy plant material	-	19.09	-	19.22	-	18.95
Fragaria × ananassa	Strawberry (Fruit)	Healthy plant material	-	19.73	-	19.47	-	19.41
Malus domestica	Apple	Healthy plant material	-	19.89	-	19.51	-	19.78
Prunus armeniaca	Apricot	Healthy plant material	-	21.44	-	21.16	-	21.16
Prunus avium	Cherry	Healthy plant material	-	22.44	-	22.32	-	22.35
Prunus persica	Peach	Healthy plant material	-	21.63	-	21.54	-	21.60
Prunus persica	Peach	Healthy plant material	-	13.05	-	12.95	-	12.98
Prunus salicina	Japanese plum	Healthy plant material	-	21.92	-	21.67	-	22.17
Prunus sp.	Plum	Healthy plant material	-	15.20	-	15.08	-	15.00
Pyrus communis	Pear	Healthy plant material	-	12.86	-	12.62	-	12.63
Pyrus pyrifolia var. Kosui	Asian pear	Healthy plant material	-	18.88	-	18.26	-	17.94
Pyrus pyrifolia var. Kumoi	PI 228015	Healthy plant material	-	18.25	-	18.12	-	18.49

Organism	Sample ID	DNA Source	Prunorum Assay		WI-B-T-1-116 (mali)		WI-B-T-1-66 (Phpl)	
			PE639	18S	*imp*	18S	23S	18S
Pyrus pyrifolia var. Hayatama	Asian pear	Healthy plant material	-	19.07	-	18.77	-	18.77
Pyrus pyrifolia var. Tama	Asian pear	Healthy plant material	-	17.63	-	17.23	-	17.39
Pyrus ussuriensis var. China	PI 542009	Healthy plant material	-	17.04	-	16.80	-	16.81
Pyrus ussuriensis var. Mien Suan Li	PI 542006	Healthy plant material	-	17.34	-	17.06	-	17.26
Aureobasidium pullulans	DSM 14940	Culture	-	-	-	-	-	-
Aureobasidium pullulans	DSM 14941	Culture	-	-	-	-	-	-
Erwinia billingae	Eh24	Culture	-	38.41	-	38.97	-	39.69
Pantoea agglomerans	E325	Culture	-	-	-	-	-	-
Pantoea vagans	C9-1	Culture	-	-	-	-	-	-
Pseudomonas fluorescens	A506	Culture	-	-	-	38.03	-	-
Alternaria alternata	22033001-01 (B)	Culture	-	38.54	-	38.94	-	39.91
Colletotrichum gloeospiriodes	22033001-01 (C)	Culture	-	-	-	36.70	-	-
Colletotrichum gloeospiriodes	22033001-01 (D1)	Culture	-	-	-	37.76	-	-
Colletotrichum queenslandicum	17030501 (C-5)	Culture	-	38.39	-	35.78	-	38.50
Colletotrichum queenslandicum	17030501 (C-11)	Culture	-	38.68	-	35.90	-	38.87
Erwinia amylovora	Fire blight	Culture	-	-	-	-	-	-
Erwinia amylovora	Fire blight	Infected plant material	-	21.46	-	12.79	-	21.62
Erwinia amylovora	Ea110	Culture	-	-	-	-	-	-
Erwinia amylovora	Ea153	Culture	-	-	-	-	-	-
Erwinia amylovora	Parkdale	Culture	-	-	-	-	-	-
Erwinia aphidicola	17110702-04	Culture	-	-	-	-	-	-
Erwinia billingae	NCPPB 661[T]	Culture	-	-	-	-	-	-
Erwinia persicina	LA659	Culture	-	-	-	-	-	-
Erwinia persicina	LA661	Culture	-	38.92	-	38.69	-	39.17
Erwinia persicina	19122706-01	Culture	-	-	-	-	-	-
Erwinia piriflorinigrans	CFBP 5888[T]	Culture	-	-	-	-	-	-
Erwinia pyrifoliae	Ep 28/96	Culture	-	-	-	-	-	-
Erwinia pyrifoliae	Ep 4/97	Culture	-	-	-	-	-	-
Erwinia pyrifoliae	Ejp 556	Culture	-	36.64	-	-	-	36.22
Erwinia pyrifoliae	Ejp 617	Culture	-	34.85	-	-	-	35.10
Erwinia pyrifoliae	23-02275	Culture	-	38.95	-	39.62	-	38.75
Erwinia rhapontici	19122702-01	Culture	-	-	-	-	-	-
Erwinia tasmaniensis	Et1/99[T]	Culture	-	-	-	-	-	-
Erwinia uzenensis	NCPPB 4475[T]	Culture	-	-	-	-	-	-
Monilinia fructicola	19090401-01A	Infected plant material	-	23.00	-	23.81	-	23.13
Monilinia fructicola	19090401-02A	Infected plant material	-	22.73	-	23.27	-	23.12
Monilinia fructicola	Mf35	Culture	-	36.70	-	37.39	-	37.41
Monilinia fructigena	Mfg2-GE-A E	Culture	-	38.56	-	-	-	39.25
Monilinia laxa	PSG1	Culture	-	-	-	-	-	-
Monilinia polystroma	SP61	Culture	-	-	-	-	-	-
Neofabraea alba	PD-1696	Infected plant material	-	25.08	-	25.86	-	25.10
Neofabraea sp.	PD-1597	Infected plant material	-	28.40	-	30.11	-	28.67
Neofabraea sp.	PD-1617	Infected plant material	-	24.70	-	26.14	-	24.73
Neofabraea sp.	PD-1655B	Infected plant material	-	27.75	-	28.91	-	27.75
Pantoea agglomerans	23091801-01	Culture	-	-	-	-	-	-
Pantoea agglomerans	23091801-03	Culture	-	-	-	-	-	-
Pantoea agglomerans	23091801-04	Culture	-	-	-	-	-	-
Pantoea allii	23091801-02	Culture	-	-	-	-	-	-
Pantoea ananatis	E	Culture	-	36.87	-	35.21	-	33.68
Pantoea ananatis	CES-5 (JM-55)	Culture	-	31.41	-	31.64	-	31.26
Pantoea ananatis	CES-14 (SM-272)	Culture	-	32.03	-	31.82	-	32.12
Pantoea stewartii	DCop3-07	Culture	-	36.29	-	36.02	-	36.21
Pantoea stewartii	PP685	Culture	-	34.79	-	34.96	-	35.51
Phacidiopycnis wasingtonensis	PD-1597	Infected plant material	-	28.40	-	30.11	-	28.67
Phacidiopycnis wasingtonensis	PD-1655B	Infected plant material	-	27.75	-	28.91	-	27.75
Pseudomonas syringae	JL2583	Culture	-	-	-	-	-	-
Sphaeropsis pyriputrescens	PD-1655B	Infected plant material	-	27.75	-	28.91	-	27.75
Venturia inaequalis	Apple Scab	Infected plant material	-	12.56	-	12.79	-	12.85
Xanthomonas arboricola pv. *corylina*	JL2611	Culture	-	-	-	-	-	-
Xyllela fastidiosa	XFS 253	Infected plant material	-	23.27	-	24.05	-	23.65
Xyllela fastidiosa	XFS 254	Infected plant material	-	19.71	-	19.94	-	20.00
Xyllela fastidiosa	XFS 946	Infected plant material	-	17.00	-	17.88	-	17.49
Xyllela fastidiosa subsp. *multiplex*	Peach Texas A&M (1)	Infected plant material	-	15.31	-	17.03	-	15.78
Xyllela fastidiosa subsp. *multiplex*	Peach Leaves Georgia	Infected plant material	-	13.66	-	14.31	-	14.18

[a]Late positive amplification with PE639 was observed with two strains of '*Candidatus* Phytoplasma mali' (AP-15 and APxN). Amplicon sequencing was used to determine the false results were from contamination. 16S amplicons were sequenced using MinION (Oxford Nanopore Technologies, Oxford, UK). Reads were imported into Geneious Prime and used to make a custom BLAST database. 16S sequence from the '*Ca*. P. prunorum' reference (AJ542544) was used as the query to identify reads from '*Ca*. P. prunorum.' Analyses revealed the presence of '*Ca*. P. prunorum' within these samples, suggesting contamination or coinfection. Approximately three and fifteen reads were found in the AP-15 and APxN extracts, respectively. While no direct conversion between read number and C_t value can be made, data demonstrated the appropriate correlation between read number and relative C_t value; APxN extract contained more reads and produced a lower relative C_t value that AP-15, corresponding to increased contamination.

Table S8. Repeatability data reflecting real-time PCR results for serial dilutions of DNA from '*Ca.* P. prunorum' strains ESFY1, LNp and LNS1 in healthy peach (*Prunus persica*) DNA tested by a single operator in a QuantStudio™ 5.

***Candidatus* Phytoplasma prunorum' ESFY1**										
	**PE639-FAM**					**23S-FAM**				
**Log Dilution Spike**	**Mean**	**Std Dev**	**CV (%)**	**N**	**% Positive**	**Mean**	**Std Dev**	**CV (%)**	**N**	**% Positive**
0	24.63	0.24	0.98%	3	100.00%	24.87	0.27	1.07%	3	100.00%
1	28.51	0.23	0.81%	3	100.00%	28.76	0.28	0.96%	3	100.00%
2	32.13	0.04	0.13%	3	100.00%	32.28	0.22	0.67%	3	100.00%
3	35.46	0.58	1.65%	3	100.00%	35.55	0.10	0.28%	3	100.00%
4	37.07	-	-	3	33.33%	39.75	0.11	0.27%	3	66.67%
5	-	-	-	3	0.00%	-	-	-	3	0.00%
***Candidatus* Phytoplasma prunorum' LNP**										
	**PE639-FAM**					**23S-FAM**				
**Log Dilution Spike**	**Mean**	**Std Dev**	**CV (%)**	**N**	**% Positive**	**Mean**	**Std Dev**	**CV (%)**	**N**	**% Positive**
0	25.66	0.02	0.07%	3	100.00%	26.32	0.07	0.25%	3	100.00%
1	29.06	0.05	0.16%	3	100.00%	29.89	0.23	0.76%	3	100.00%
2	32.38	0.16	0.49%	3	100.00%	33.18	0.23	0.70%	3	100.00%
3	36.35	0.55	1.51%	3	100.00%	35.96	0.61	1.70%	3	100.00%
4	37.49	-	-	3	33.33%	38.45	-	-	3	33.33%
5	-	-	-	3	0.00%	-	-	-	3	0.00%
***Candidatus* Phytoplasma prunorum' LNS1**										
	**PE639-FAM**					**23S-FAM**				
**Log Dilution Spike**	**Mean**	**Std Dev**	**CV (%)**	**N**	**% Positive**	**Mean**	**Std Dev**	**CV (%)**	**N**	**% Positive**
0	22.76	0.20	0.87%	3	100.00%	25.07	0.21	0.84%	3	100.00%
1	26.03	0.11	0.43%	3	100.00%	28.01	0.12	0.44%	3	100.00%
2	29.40	0.14	0.48%	3	100.00%	31.25	0.15	0.48%	3	100.00%
3	32.39	0.26	0.80%	3	100.00%	34.35	0.23	0.66%	3	100.00%
4	35.98	0.73	2.04%	3	100.00%	37.85	0.27	0.71%	3	66.67%
5	37.63	-	-	3	33.33%	-	-	-	3	0.00%

Mean = average cycle threshold (C_t) value
Std Dev = standard deviation
CV = coefficient of variation (Std Dev / Mean)

Table S9. Intermediate precision data reflecting real-time PCR results for 10-fold serial dilutions serial dilutions of DNA from '*Ca.* P. prunorum' strain ESFY1 in healthy peach (*Prunus persica*) DNA tested by three independent operators in different QuantStudio™ 5 instruments.

**Levels of Variation:** *Three different operators ; different ABI QS5s*					
**Log Dilution Spike**	**Mean**	**Std Dev**	**N**	**CV (%)**	**% Positive**
0	25.09	0.39	9	1.55%	100.00%
1	28.92	0.34	9	1.17%	100.00%
2	32.38	0.19	9	0.60%	100.00%
3	35.81	0.39	9	1.09%	100.00%
4	38.17	0.97	9	2.55%	33.33%
5	-	-	9	-	0.00%
			**AVERAGE**	**1.10%**	

Mean = average cycle threshold (C_t) value
Std Dev = standard deviation
CV = coefficient of variation (Std Dev / Mean)

Table S10. Reproducibility data reflecting real-time PCR results for 10-fold serial dilutions of DNA from '*Ca.* P. prunorum' strain ESFY1 in healthy peach (*Prunus persica*) DNA tested by three independent operators and two different instruments (QuantStudio™ 5 and CFX OPUS).

**Levels of Variation:** *Three operators ; ABI QS5s and Bio-Rad CFX OPUS*					
**Log Dilution Spike**	**Mean**	**Std Dev**	**N**	**CV (%)**	**% Positive**
0	25.02	0.40	12	1.59%	100.00%
1	28.79	0.37	12	1.29%	100.00%
2	32.35	0.17	12	0.54%	100.00%
3	35.86	0.46	12	1.29%	100.00%
4	38.01	0.86	12	2.26%	33.33%
5	-	-	12	-	0.00%
			**AVERAGE**	**1.18%**	

Mean = average cycle threshold (C_t) value
Std Dev = standard deviation
CV = coefficient of variation (Std Dev / Mean)

Table S11. Reaction metrics determined by testing serial dilutions of DNA from '*Ca*. P. prunorum' strains ESFY1, LNp and LNS1 with the PE639 and 23S assays, and '*Ca*. P. mali' strains C71, P4 and P6 with the *imp* and 23S assays.

***Candidatus Phytoplasma* prunorum'**								
	**PE639-FAM**				**23S-FAM**			
**Strain**	**N**	**R^2**	**Slope**	**Amp. Eff.**	**N**	**R^2**	**Slope**	**Amp. Eff.**
**ESFY1**	12	0.9922	-3.6083	89.29%	3	0.9963	-3.5561	98.61%
**LNp**	3	0.9947	-3.5369	91.75%	3	0.9908	-3.2210	104.39%
**LNS1**	3	0.9953	-3.2799	101.78%	3	0.9978	-3.1057	109.89%
***Candidatus* Phytoplasma mali' C71**								
	***imp*-FAM**				**23S-FAM**			
**Strain**	**N**	**R^2**	**Slope**	**Amp. Eff.**	**N**	**R^2**	**Slope**	**Amp. Eff.**
**C71**	12	0.9911	-3.4352	95.48%	3	0.9900	-3.3512	98.79%
**P4**	3	0.9905	-3.1402	108.19%	3	0.9908	-3.1487	107.77%
**P6**	3	0.9957	-3.4965	93.20%	3	0.9972	-3.2124	104.78%

Amp. Eff. = amplification efficiency = $(10^{\frac{-1}{\text{slope}}} - 1) \times 100\%$

Table S12. Data from stability testing of the synthetic gBlocks™ positive control working dilution (4 fg/µl) in TE buffer with 0.5 mg/ml polyadenylic acid (poly(A) using PE639 and 23S assays.

Levels of Variation: *Three different operators ; different ABI QS5s; different aliquots; varying freeze-thaw*

Aliquot/Freeze-Thaw	N	FAM - PE639 Mean	FAM - PE639 Std Dev	FAM - PE639 CV (%)	FAM - PE639 % Positive	ABY - 18S Mean	ABY - 18S Std Dev	ABY - 18S CV (%)	ABY - 18S % Positive
**Operator 1**									
1	3	25.07	0.59	2.34%	100.00%	28.04	0.20	0.70%	100.00%
2	3	25.36	0.11	0.42%	100.00%	28.02	0.19	0.69%	100.00%
3	3	25.41	0.06	0.25%	100.00%	27.89	0.16	0.57%	100.00%
4	3	25.37	0.07	0.26%	100.00%	27.90	0.17	0.60%	100.00%
5	3	25.28	0.16	0.65%	100.00%	27.72	0.24	0.85%	100.00%
			**AVERAGE**	**0.78%**			**AVERAGE**	**0.68%**	
**TOTAL (N=15)**		25.30	0.27	1.06%		27.91	0.20	0.72%	
**Operator 2**									
1	3	25.68	0.04	0.16%	100.00%	28.16	0.07	0.23%	100.00%
2	3	25.73	0.03	0.14%	100.00%	28.08	0.05	0.17%	100.00%
3	3	25.59	0.02	0.09%	100.00%	27.93	0.03	0.12%	100.00%
4	3	25.62	0.05	0.21%	100.00%	27.88	0.03	0.11%	100.00%
5	3	25.60	0.09	0.34%	100.00%	27.91	0.03	0.11%	100.00%
			**AVERAGE**	**0.19%**			**AVERAGE**	**0.15%**	
**TOTAL (N=15)**		25.64	0.06	0.23%		27.99	0.11	0.40%	
**Operator 3**									
1	3	25.55	0.02	0.07%	100.00%	27.94	0.12	0.41%	100.00%
2	3	25.57	0.05	0.19%	100.00%	27.90	0.19	0.68%	100.00%
3	3	25.48	0.09	0.35%	100.00%	27.82	0.23	0.82%	100.00%
4	3	25.59	0.04	0.15%	100.00%	27.82	0.16	0.58%	100.00%
5	3	25.47	0.09	0.35%	100.00%	27.78	0.15	0.55%	100.00%
			**AVERAGE**	**0.22%**			**AVERAGE**	**0.61%**	
**TOTAL (N=15)**		25.53	0.07	0.29%		27.85	0.16	0.57%	
**Combined TOTAL (N=45)**		25.49	0.22	0.85%		27.92	0.17	0.60%	

Mean = Average cycle threshold (C_t) value
Std Dev = standard deviation
CV = coefficient of variation (Std Dev / Mean)

Table S13. Cost calculation for the synthetic gBlocks control, not reflective of operational costs.

Phytoplasma gBlocks synthetic positive control										
**Reagent**	**Vendor**	**Cat. No.**	**Amount**	**Cost**	**Working Amount**	**Working Volume**	**Amount/rxn**	**Rxns/container**	**Cost/rxn**	**Cost/1000 rxns**
gBlocks Gene Fragments 1751-2000 bp	IDT	NA	100 µl @ 10 ng/µl	$397.00	2.5E8 µl @ 4 fg/µl	250000000	2	125000000	$0.00	$0.00
TE buffer	Promega	V6231	100 ml	$38.51	100000 µl	100000	2	50000	$0.00	$0.77
poly(A)	Millipore Sigma	10108626001	100 mg	$187.00	0.5 mg/ml TE	200000	2	100000	$0.00	$1.87
								**Subtotal:**	$0.00	$2.64
General Consumables										
Add 20% for tips, tubes, gloves, and other common supplies.								**Total:**	$0.00	$0.53
.										
**Company**	**Type**	**Base Cost**	**Limiting Rxns**						**Cost/rxn**	**Cost/1000 rxns**
Millipore Sigma	Shipping	$35.00	100000						$0.00	$0.35
IDT (per order if less than 12 primers)	Shipping	$3.50	125000000						$0.00	$0.00
								**Total:**	$0.00	$0.35
								**Grand Total:**	$0.00	$3.52
